# Weight Care Project: Health professionals' attitudes and ability to assess body weight status - Study protocol

**DOI:** 10.1186/1471-2458-11-202

**Published:** 2011-03-31

**Authors:** Anne Moorhead, Vivien Coates, Diane Hazlett, Alison Gallagher, Kathy Murphy, Geraldine Nolan, John Dinsmore

**Affiliations:** 1 School of Communication, University of Ulster, Shore Road, Newtownabbey, BT37 0QB, Northern Ireland; 2 School of Nursing, University of Ulster, Cromore Road, Coleraine, BT52 1SA, Northern Ireland; 3 School of Biomedical Sciences, University of Ulster, Cromore Road, Coleraine, BT52 1SA, Northern Ireland; 4 School of Nursing, National University of Ireland, Galway Republic of Ireland; 5 Department of Health Promotion, National University of Ireland, Galway Republic of Ireland

## Abstract

**Background:**

Health professionals working in primary care and public health have opportunities to address body weight status issues with their patients through face-to-face contact. The objectives of this all-Ireland project are: 1. to assess the attitudes, current practices/behaviours and knowledge of key health professional groups on body weight status; 2. to assess the health professional groups' ability to identify body weight status in both adults and children. The health professional groups are: (a) community related public health nurses; (b) school public health nurses; (c) GPs and practice nurses (primary care); and (d) occupational health nurses (workplace) from both Northern Ireland and the Republic of Ireland.

**Methods/Design:**

This all-Ireland multi-disciplinary project follows a mixed methods approach using both quantitative and qualitative methodologies, and consists of four components:

1. Literature review - to explore the role of health professionals in managing obesity through spontaneous intervention in a variety of health promotion settings.

2. Telephone interviews and focus groups - to gain an in-depth insight into the views of health professionals in assessing body weight status.

3. Survey (primarily online but also paper-based) - to determine the attitudes, current practices/behaviours and knowledge of health professionals in assessing body weight status.

4. Online evaluation study - an online interactive programme will be developed to assess health professionals' ability to identify the body weight status of adults and children.

**Discussion:**

This project will assess and report the attitudes, current practices/behaviours and knowledge of key health professional groups within Northern Ireland and the Republic of Ireland on body weight status, and their ability to identify body weight status in both adults and children. The results of this project will generate recommendations for clinical practice in managing obesity, which may inform policy guidelines.

## Background

Obesity is a growing global epidemic [1] and Ireland is no exception with obesity in Irish adults increasing by at least 1% every year [2]. Data from The North/South Ireland Food Consumption Survey observed that on an all-Ireland basis 39% of the adult population were overweight and 18% were obese [3]. This is supported by the SLÁN Survey 2007 [4,5] in the Republic of Ireland. Data from children indicated that almost one in four boys and over one in four girls were either overweight or obese in both Northern Ireland and the Republic of Ireland [6]. The Report of the National Taskforce on Obesity [2] also highlighted the importance of managing obesity in Ireland and provided over 80 recommendations. Therefore, obesity is a key public health issue within Ireland.

There is an increasing body of evidence that large proportions of adults fail to identify themselves and/or their children as overweight or obese [7-10]. Furthermore, this may place responsibility on health professionals to assess body weight status. However, there is limited published research of health professionals assessing body weight status. A US study has shown that 25% of GPs failed to recognise their overweight patients [11]. Studies in the UK indicate that GPs may have a negative attitude towards obese patients [12,13], and further studies have also reported this among physicians [14] and a range of health professionals [15]. Nevertheless, health professionals could have a vital role in initiating recognition of body weight status among the population, which may lead to behaviour changes in the prevention of obesity.

At present, no published research data exists on the attitudes of health professionals towards assessing body weight status in either Northern Ireland or the Republic of Ireland. Furthermore, it is not known whether, when, how regularly nor the accuracy of health professionals throughout Ireland assessing body weight status, and also their ability to identify body weight status of adults and children. Thus the objectives of this all-Ireland multidisciplinary project are: 1. to assess the attitudes, current practices/behaviours and knowledge of key health professional groups on body weight status; 2. to assess the health professional groups' ability to identify body weight status in both adults and children. Targets groups were chosen as they are the key health professionals with opportunities to address body weight issues with their patients. These groups include: community related public health nurses; school public health nurses; GPs and practice nurses (primary care); and occupational health nurses (workplace). For the purposes of this project, body weight categories (underweight, normal weight, overweight, obese) are defined according to the World Health Organisation's (WHO) BMI classification for adults [16] and children [17,18]. This project is known as the Weight Care Project.

## Methods/Design

This project employs a mixed methods approach using both quantitative and qualitative methodologies, and consists of four components: 1. literature review; 2. telephone interviews and focus groups; 3. survey; 4. online evaluation study. All the four target groups will be included in each data collection component.

### Recruitment

Several strategies will be utilised to maximise participant recruitment. Health professionals will be invited to participate in this project via articles published in their professional organisations/associations' bulletins/newsletters. The researchers will access events in Northern Ireland and the Republic of Ireland where nurses and GPs will attend to invite them to participate in this project, this will include the following: health trust staff meetings; special interest groups; classes with postgraduate nurses at the researchers' universities; conferences and workshops. Interested health professionals will contact the researchers and will be screened for the selection criteria. The inclusion criteria include: currently employed in one of the target clinical areas (as reported by the participants) and willing to participate. The exclusion criterion will be not able to provide informed consent. Eligible participants will provide informed consent. A project website was developed to support the project and will provide information for participants including e-mail address, telephone mailbox, information sheets, and will host the online survey and online evaluation study (http://www.science.ulster.ac.uk/weightcareproject).

### 1. Literature review

The purpose of the literature review is to explore the role of health professionals in managing obesity through spontaneous intervention in a variety of health promotion settings. Although the gold standard review is a systematic review, which includes only randomised controlled trials (RCTs), for the purposes of this project and to achieve the comprehensive relevant literature, a systematic approach will be adopted to include studies with all designs. A systematic search of the literature to locate studies will be conducted using the following electronic databases: CSA Illumina, Cochrane Library, Communication Abstracts, EBSCO Host CINAHL, ISI Web of Knowledge: Web of Science, OvidSP Embase, OvidSP Medline, OVIDSP PsychINFO, and PubMeb Central (within the last 10 years, 2000 to 2010). To obtain the comprehensive relevant literature, no studies will be excluded on the basis of study design, outcome measures or type of intervention. Study quality will be critiqued. References from the articles will also be reviewed. The findings from this review process will contribute to and confirm the methodologies of this project.

### 2. Telephone interviews and focus groups

The purpose of the telephone interviews and focus groups are to gain in-depth insight into the views of health professionals regarding the attitudes, current practices/behaviours and knowledge of assessing body weight status. These discussions will be centred on attitudes, current practices/behaviours and knowledge of health professionals in assessing body weight status amongst their patients.

#### Study design

Quantitative and qualitative methodologies will be employed using quantitative telephone semi-structured interviews with GPs and focus groups with nurses. This design is based on the researchers' experiences of obtaining in-depth information from GPs and nurses.

#### Sample size

A stratified convenience sample size will be used but will ensure representation from both Northern Ireland and Republic of Ireland. In total there will be 16 focus groups conducted. There were approximately 84,922 registered nurses actively practicing throughout Ireland in 2008 with 68,614 in the Republic of Ireland [19] and 16,308 in Northern Ireland [20,21]. According to the Nursing Board registered statistics for 2008, there are 3,040 public health nurses in the Republic of Ireland [19]. The numbers of doctors registered on the specialist division of General Practice (GP) in 2008 was 1626 in the Republic of Ireland [22] and 1156 in Northern Ireland [23]. At the time of writing, there were 35 Clinical Nurse Specialist posts approved in Occupational Health and one Advanced Nurse Practitioner post approved in Occupational Health in the Republic of Ireland [24]. The only figures available for occupational health nurses in Northern Ireland is that there are two currently registered as members with The Association of Occupational Health Nurse Practitioners (UK) [25]. To obtain approximate proportional representation, 12 focus groups will be conducted in the Republic of Ireland and four in Northern Ireland. There will be eight participants in each focus group, thus 128 nurses are required to participate in the focus groups. This meets the recommendation that a focus group should consist of between four to eight participants [26]. For each focus group, 12 participants will be recruited to achieve at least eight participants, thus a total of 192 nurses will be recruited. Telephone interviews will be conducted with 16 GPs (8 from Northern Ireland and 8 from the Republic of Ireland).

#### Procedures

A questionnaire will be developed for the telephone interviews with GPs, which will be quantitative-based. These interviews will be conducted at a time of the GP's convenience. Focus groups will be conducted during/after conferences/workshops and staff meetings in the health clinics/centres, hospitals or universities, convenient for the nurses. Example questions for both the interviews and focus groups will include 'outline your experience of working with people who are overweight/obese?' and 'what are the ways which you measure body weight status and also what have been the issues in using these measures? Different scenarios of overweight or obese adults and children will be discussed. All focus groups will be recorded.

The themes and issues from the interviews and focus groups will be collated and then explored further via developing a questionnaire for the survey. A selected sample of the participants from focus groups will meet on a second occasion to pilot the survey.

### 3. Survey

This survey aims to determine the attitudes, current practices/behaviours and knowledge of assessing body weight status among health professionals in Northern Ireland and the Republic of Ireland.

#### Study design

Survey using a questionnaire, primarily online but also will be made available as required in paper-based format.

#### Sample size

From previous research by a team member (KM), the usual response rate for online surveys with health professionals varies but is approximately 50%. Sample size was calculated using a confidence level of 95%, confidence Interval of 5 with the target population of approximately 88000 in total (previously described), giving a sample size of 382. Based on an estimated 50% response, 764 participants will be recruited for this survey. GPs and nurses will be recruited proportionally within Republic of Ireland and Northern Ireland (i.e. 4:1 for nurses and also for GPs, 1:1, respectively).

#### Questionnaire

The questionnaire will ask the health professionals questions on body weight status amongst both adults and children. This will include: the current views on assessing body weight status; the extent to which body weight status is currently assessed, and if assessed how it is measured (tools/scales), how accuracy is determined/assessed; and the health professionals' understanding of what constitutes healthy and overweight/obese body weight statuses; and the extent of continuous professional development. The duration of years of clinical experience (date of qualification) will be recorded to determine if there are any differences in years of experience. In addition, issues will be explored relating to how health professionals communicate obesity messages in managing obesity with other health professionals and with patients (adults and children including parents, and their families).

#### Procedures

Health professionals will complete the questionnaire on one occasion. Health professionals that do not have access to the internet and who indicate willingness to participate in this project will be forwarded a hard copy of the questionnaire by post. In addition, the researchers will go along to meetings/conferences/workshops attended by the health professionals and will ask them to complete the questionnaire (online or hard copy) during and/or at the end of the event.

### 4. Online evaluation study

The primary objective of the online evaluation study is to determine if health professionals can identify the body weight status of adults and children correctly and if there is a significant difference between the responses from the health professionals and the expert responses (i.e. researchers' responses).

#### Study design

Online evaluation study consisting of a novel approach of a video of images with different body weight statuses as a basis for a series of questions.

#### Sample size

For purposes of feasibility and limited time, a sample size for one group was calculated, which included all the target health professionals groups in both Northern Ireland and the Republic of Ireland. This sample size was calculated using GPower 3.0.10 [27] with the level of significance set at 0.05 and 80% power using a 2-tailed t-test. This was calculated using data from a US study that 25% of GPs failed to recognise their patients were overweight [11]. The power calculation indicated a total sample size of 128 participants. Taking into account a drop-out rate of approximately 20%, in total at least 154 participants (nurses and GPs) will be recruited across Ireland.

#### Development and validation of DVD/video

For the purposes of this project, a DVD/video will be developed, which will be used to assess the ability of health professionals in Northern Ireland and the Republic of Ireland to recognise overweight and obesity in adults and children. The DVD/video will consist of series of 20 case studies (adults and children), which each image will rotate by 360°. The DVD/video approach was chosen instead of a validated method such as silhouettes because it represents an innovative tool, which will be both a practical and feasible method for health professionals to assess body weight within the allocated timeframe. The DVD/video will aim to construct a real-life health professional-patient consultation within Ireland. The DVD/video will be interactive, with movement, and based on real people, features that have not been included in previous methods. The case studies will be based on volunteer models of various ages (children <18 years; adults 18-65 years; older adults >65 years), gender (female/male), body weights and heights to provide a range of BMIs. Body weight status will be based on the WHO's BMI classification [14] (Underweight, BMI < 19.9 kg/m^2^; normal weight, BMI 20-25.0 kg/m^2^; overweight, BMI 25.1 - 29.9 kg/m^2^; obese BMI >30.0 kg/m^2^) with the International Obesity Taskforce (IOTF) cut-offs for BMI [17,18] being used to determine body weight category for case studies <18 years.

A pilot/validation online evaluation study in a form of a focus group will be conducted prior to the main online evaluation study. The aim of this validation/pilot study is to determine if the online study is functioning properly and collecting the required data, checking for timing, clarity and nagaviation of the videos and questions, and to compare the new tool with an existing validated silhouette tool. Within the focus group, a convenience sample of 30 (20% of main study) health professionals will complete the online evaluation study and then provide feedback to the researchers. The responses from the health professionals i.e. measurements from the real-life case studies will be compared with the validated silhouettes (using paired t-tests, SPSS) to determine if there are any significant differences between the two tools.

#### Procedures

Participants will complete the online evaluation study on one occasion. This will consist of the participants being shown each case study one at a time and in a random order and will be asked to complete a series of questions. Participant responses will be assessed to determine the extent to which health professionals are able to identify overweight/obesity and questions will be included, which will also evaluate their understanding of how to assess overweight/obesity. It is envisaged that it will take participants no more than 45 minutes to complete this online evaluation study.

To ensure that each of the target health professional groups from both Northern Ireland and the Republic of Ireland are represented, those who do not have internet access will have an opportunity to complete this online study at meetings/conferences/workshops. The researchers will attend meetings/conferences/workshops with laptops for participants to complete this online evaluation study.

### Data analysis

The recordings from the focus groups will be transcribed independently. The data generated from the focus groups will be analysed and responses will be grouped into themes and key issues using content analysis of the transcripts [28-30].

Quantitative data from the interviews, online survey and online evaluation study will be analysed using SPSS. Summary statistics (descriptive and frequencies) and inferential statistics will be conducted, as appropriate. Following the test for normality, if showing normal distribution of data, t-tests/analysis of variance and correlations will be conducted to determine any significances and relationships in the data. Paired t-tests will be conducted using the data from the online evaluation study to determine if there is a significant difference between the responses from the health professionals and the expert responses. If the assumptions for the above parametric tests are violated then the appropriate non-parametric tests such as the Mann-Whitney will be used.

### Ethical considerations

As this is an all-Ireland project, ethical approval was obtained from six ethical committees in both Northern Ireland and the Republic of Ireland, these were: School of Communication, University of Ulster; Office for Research Ethics Committees Northern Ireland (ORECNI); Belfast Health and Social Care Trust; National University of Ireland, Galway; University College Hospital Galway; Irish College of General Practitioners. There is a Project Steering Committee who will monitor this project.

The key ethical issues in this project are informed consent, participant's confidentiality and data protection, as according to good clinical practice guidelines. All the participants in each study will receive information following an appropriate cooling-off period (at least one week) and with full understanding provide informed consent. Participants' privacy and confidentiality will be maintained during the data collection and all participants' information will be treated confidential as per the Data Protection Act (1998) [31]. To protect participants' confidentiality, each participant will be assigned a study identification (ID) number. The online questionnaires will be accessed using passwords provided.

Written protocols for (a) management of distress should the models, particularly the child models, become distressed; (b) recording of and safeguard of the distribution of the DVD/video; and (c) filming procedures have been compiled. Each model will be filmed individually. The filming will be conducted in a confidential and comfortable setting.

## Discussion

This all-Ireland multidisciplinary Weight Care Project aims to assess the attitudes, current practices/behaviours and knowledge of key health professional groups in Northern Ireland and the Republic of Ireland on body weight status; and also to assess the ability of health professional groups to identify body weight status in both adults and children. The target health professional groups include: community related public health nurses; school public health nurses; GPs and practice nurses (primary care); and occupational health nurses (workplace). This will be achieved using a range of qualitative and quantitative research methodologies, namely interviews, focus groups, survey and an online evaluation study. The DVD/video incorporated into an online evaluation study is an innovative method to assess the health professional groups' ability to identify body weight categories in both adults and children. A range of strategies will be employed to maximise recruitment of nurses and GPs.

As there is limited published research of health professionals assessing body weight status, this project will contribute knowledge to this area. This project will determine the attitudes of health professionals towards assessing body weight status; and whether, when, and how regularly health professionals assess body weight status, and how accurate health professionals are in assessing body weight status of adults and children. In addition, issues relating to how health professionals communicate obesity messages in managing obesity with other health professionals and with patients (adults and children including parents, and their families) will be reported. This information may contribute to enhancing the communication of obesity messages within healthcare.

The findings from this project may contribute to reducing a key global public health issue of obesity among adults [2-5] and children [6]. This project should increase the awareness of overweight and obesity among health professionals, which previous studies reported that GPs (25%) failed to recognise their patients were overweight [11], and may have a negative attitude towards obese patients [12-15]. Health professionals could have a vital role in initiating recognition of body weight issues among the population, and may help initiate positive behaviour changes in the prevention of obesity. This research will contribute to other work in Northern Ireland and the Republic of Ireland to increase awareness of obesity and encourage behaviour change to reduce the prevalence of obesity among Irish adults and children and potentially globally. The results of this project will generate recommendations for clinical practice in managing obesity, which may inform policy guidelines.

## Competing interests

The authors declare that they have no competing interests.

## Authors' contributions

AM designed the project methodology, Principal Investigator for this project, and drafted the first version of this manuscript. VC, DH, AG, KM, GN contributed to the development of the research protocol and were co-investigators on the successful funding application. All authors contributed to all versions and approved the final manuscript.

## Pre-publication history

The pre-publication history for this paper can be accessed here:

http://www.biomedcentral.com/1471-2458/11/202/prepub
